# Surgical Repair of Subacute Right Ventricular Perforation after Pacemaker Implantation

**DOI:** 10.1155/2017/3242891

**Published:** 2017-04-06

**Authors:** Takeshi Oda, Takanori Kono, Keiichi Akaiwa, Yasushi Takahara, Chie Yasuoka, Katsuhiko Nakamura

**Affiliations:** ^1^Department of Cardiovascular Surgery, Omura Municipal Hospital, 133-22 Kogashima-machi, Omura, Nagasaki 856-8561, Japan; ^2^Department of Cardiology, Omura Municipal Hospital, 133-22 Kogashima-machi, Omura, Nagasaki 856-8561, Japan

## Abstract

We report an 84-year-old woman who presented with right ventricular perforation 4 days after pacemaker implantation for syncope due to sick sinus syndrome. Median sternotomy revealed no pericardial effusion, but the pacing lead had penetrated the right ventricle and pericardium. When the pleura was opened, the tip of the lead was seen in the visceral pleura. The lead was cut in the pericardial cavity and extracted from the left subclavian wound together with the generator. The right ventricular perforation was sutured and a temporary pacing lead was placed on the right ventricular wall intraoperatively. Ten days after the surgery, a new pacemaker lead was placed in the ventricular septum via the right axillary vein. Right ventricular perforation is a rare complication after pacemaker implantation. Typically, it occurs at the time of implantation or within 24 hours after implantation. In the present case, the perforation of the right ventricle which needed urgent surgery occurred 4 days after implanting the pacing lead at the right ventricular apex. Great care should have been taken not to overlook this life-threatening complication even more than 24 hours after pacemaker implantation.

## 1. Introduction

Cardiac perforation by a pacing lead is rare, but life-threatening complication of pacemaker implantation usually presents within 24 hours of implantation and is uncommon after that. This complication tends to be more frequent when the tip of the pacing lead is placed at the right ventricular (RV) apex rather than the ventricular septum [1]. Independent predictors of cardiac perforation after pacemaker implantation were reported to be the use of a temporary pacemaker, helical screw-in leads, and oral steroid therapy [2]. The options for treating this complication can be divided into surgical or percutaneous therapy. We report an elderly woman who presented with RV perforation by the pacing lead. After the lead was extracted surgically, the tip of a new permanent pacemaker lead was placed at the ventricular septum via the right axillary vein.

## 2. Case Report

**Figure 1:**
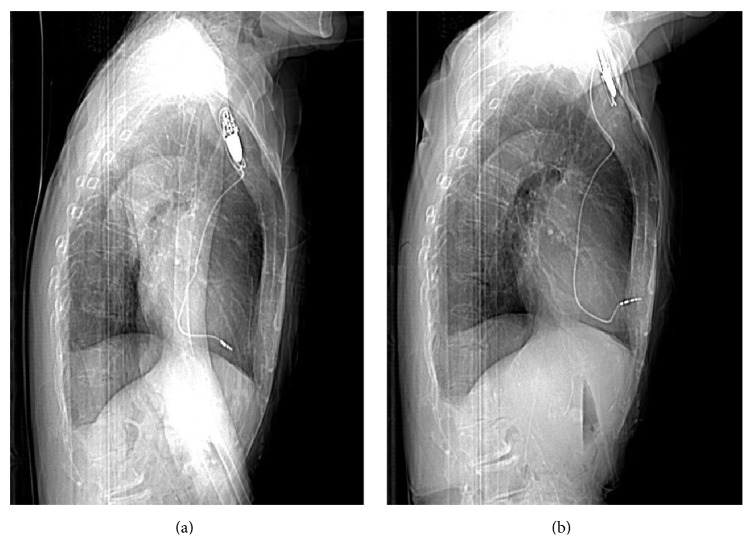
Lateral chest X-ray film demonstrating the position of the lead. (a) Just after the surgery. The tip of the lead was in the correct position. (b) Four days after the surgery. The tip of the lead intruded into the chest wall.

**Figure 2:**
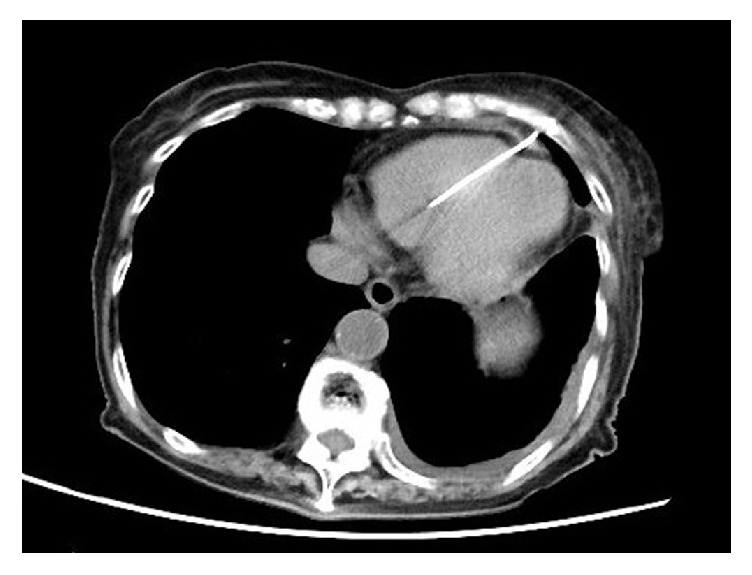
Chest CT reveals perforation of the right ventricle by the lead.

**Figure 3:**
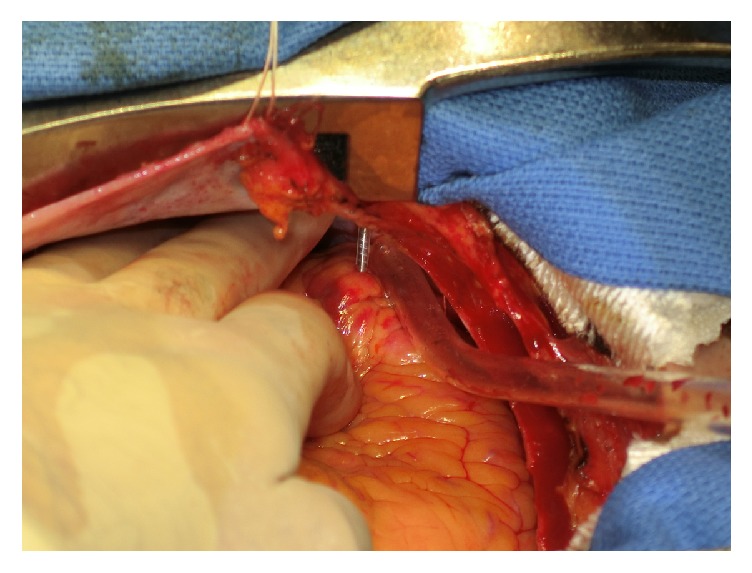
Intraoperative view of the lead penetrating the right ventricular apex.

An 84-year-old woman with a history of syncope was referred to a general hospital by her primary doctor. Sick sinus syndrome was diagnosed and a ventricular demand pacemaker was implanted via the left axillary vein. The tip of the implanted lead (5076-52 cm, Medtronic, Minneapolis, MN) was a screw type. The lateral chest X-ray film obtained just after pacemaker implantation demonstrated that the tip of the lead was in the correct position (Figure 1(a)) and the pacemaker threshold was normal. Two days after implantation, she complained of the sudden onset of pain in the left precordial region. On the fourth day after implantation, a bulge appeared in the left sixth intercostal space at the site of the pain. A lateral chest radiograph obtained on the same day demonstrated displacement of the pacemaker lead (Figure 1(b)). Transthoracic echocardiography did not identify a pericardial effusion, but the position of the lead tip could not be seen accurately. Computed tomography (CT) confirmed penetration of the lead though the RV into the subcutaneous tissues of the left thoracic cavity (Figure 2). There was no pericardial effusion, pneumothorax, or pleural effusion. The patient was sent to our hospital for emergency treatment. Because transvenous removal of the pacing lead was thought to be risky, surgical removal and RV repair were performed. Median sternotomy was chosen as the approach because it would be easy to establish cardiopulmonary bypass if required. There was no pericardial effusion, but the pacing lead was seen protruding through the anterior wall of the RV apex and penetrating the pericardium (Figure 3). When the left pleural cavity was opened, the pacing lead was found to be embedded in the anterior visceral pleura, but the left lung was not injured in agreement with preoperative CT findings. A purse string suture was placed around the lead in the RV wall and a pledgetted mattress suture was added for reinforcement. Then the part of the lead protruding from the RV was grasped with forceps and cut in the pericardial cavity. Next, the pacing lead and generator were extracted from the left subclavian wound without any resistance. At the same time, the purse string suture and then the mattress suture were tied in this order, and the remaining part of the lead was extracted from the pleura with little resistance. There was no bleeding at the site where the lead had pierced the pleura. A temporary pacing wire was placed on the RV wall intraoperatively. Ten days after surgery, a new permanent pacemaker was inserted, with the tip of the lead being placed at the ventricular septum via the right axillary vein without any problems.

## 3. Discussion

The incidence of perforation of the heart by the pacing lead after pacemaker implantation ranges from 0.1% to 0.8% [3]. While this complication is rare, it may be fatal if discovered too late. Most perforations manifest within 24 hours after pacemaker implantation [4]. In the present patient, the tip of the lead was correctly positioned in the lateral chest X-ray film obtained just after implantation, but the lead tip had obviously advanced outside the heart by the fourth day. Symptoms of cardiac perforation vary, including extracardiac muscle stimulation, chest pain, shortness of breath due to pneumothorax, and hemothorax, hemopneumothorax, pneumomediastinum, and/or pericardial tamponade [5]. Our patient started to complain of anterior chest pain on the second day after implantation, suggesting that the tip of the lead had gradually advanced to reach the pleura on that day. At surgery, the screw tip was found embedded in the intercostal subcutaneous tissue. If treatment had been delayed further, the skin might have been penetrated, resulting in infection.

There are two options for the management of ventricular perforation by a pacemaker lead, which are surgical or transvenous procedures. According to a review of 25 patients by Refaat et al. [5], the lead was extracted surgically in 14 cases and was managed transvenously in 11 cases. Severe complications occurred in two of the 11 patients receiving transvenous management, with one patient dying 10 days after lead extraction [6] and the other developing pericardial tamponade after transvenous lead extraction that required pericardiocentesis [7]. Although surgical extraction is more invasive than transvenous management, the surgical option seems to be safer. When surgery is performed, median sternotomy is more common than left anterior thoracotomy as the approach. Indeed, the left anterior thoracotomy is less invasive and has the advantages in avoiding mediastinitis or adhesion after the operation. However, we chose median sternotomy because it is the best approach for repairing RV perforation and damage to adjacent structures, we believe. It is also easy to establish cardiopulmonary bypass in an emergency when median sternotomy is chosen.

Amara et al. reported that there was a higher risk of cardiac perforation in thin elderly female patients, as well as patients on anticoagulants or steroids [7]. Our case was a thin elderly female patient, but she was not taking anticoagulants or steroids.

A change in the pacing threshold can be a sign of RV perforation by the pacemaker lead. While a chest X-ray film is convenient for detecting displacement of the lead, CT should subsequently be performed for accurate diagnosis if RV perforation seems likely.

To reduce the risk of RV perforation, placing the tip of the lead at the ventricular septum is recommended rather than implantation in the RV apex or free wall. This is because the ventricular septum is typically thicker than either the RV apex or free wall, and even if a pacing lead penetrates the septum the tip will remain in the left ventricular chamber.

## 4. Conclusion

RV perforation is a rare, but potentially fatal, complication of pacemaker implantation. It usually manifests within 24 hours, but our patient presented four days after implantation. This case emphasizes that careful postoperative observation is necessary, even if the pacing lead is positioned correctly. To avoid RV perforation, implantation of the pacemaker lead at the ventricular septum seems to be safer than selecting the RV apex or free wall.
